# Traditional Chinese medicine Lingguizhugan decoction treating non-alcoholic fatty liver disease with spleen-yang deficiency pattern: Study protocol for a multicenter randomized controlled trial

**DOI:** 10.1186/s13063-020-04362-7

**Published:** 2020-06-10

**Authors:** Jingjuan Xu, Ruirui Wang, Shengfu You, Lei Zhang, Peiyong Zheng, Guang Ji, Baocheng Liu

**Affiliations:** 1 Shanghai Innovation Center of TCM Health Service, Shanghai University of Traditional Chinese Medicine, No. 1200 Cailun Road, Shanghai, 201203 China; 2 Institute of Digestive Diseases, Longhua Hospital, Shanghai University of Traditional Chinese Medicine, No. 725 South Wanping Road, Shanghai, 200032 China

**Keywords:** Non-alcoholic fatty liver disease, Traditional Chinese medicine, Lingguizhugan decoction, Randomized controlled trial

## Abstract

**Background:**

Non-alcoholic fatty liver disease (NAFLD) is a common chronic liver disease characterized by excessive fat accumulation in the liver. One of the underlying pathophysiological mechanisms is insulin resistance (IR). Traditional Chinese medicine (TCM) has showed potential benefits in the management of NAFLD. Lingguizhugan decoction (LGZG) is a representative Chinese herbal formula; however, there is still no rigorous clinical trial supporting its application.

**Methods/design:**

This study will be a three-arm, dose-optimization, randomized, double-blinded, placebo-controlled clinical trial. A total of 243 patients with NAFLD will be recruited and randomly assigned to the standard dose LGZG (SLGD) group, low dose LGZG (LLGD) group, or the placebo group based on a ratio of 1:1:1. The treatment period will be 12 weeks and the follow-up period will last 4 weeks. The primary outcome will be the proportions of participants with at least a 1-unit decrease of HOMA-IR from baseline to 12 weeks. Secondary outcomes will include the changes of body weight, body mass index, liver function, blood lipid metabolism, blood glucose metabolism, inflammatory responses, liver-kidney echo ratio by ultrasound, and various scales. Biological samples will also be collected for future researches on mechanism exploration.

**Discussion:**

This study will provide initial evidence regarding the efficacy and safety of LGZG in the treatment of NAFLD with spleen-yang deficiency pattern and promote its application in community healthcare centers. In addition, potential mechanisms will be explored based on studies of oral and gut microbiota.

**Trial registration:**

Chinese Clinical Trial Registry, ChiCTR1800014364. Registered on 1 January 2018. The final protocol version was V3.0.

## Background

Non-alcoholic fatty liver disease (NAFLD) is one of the chronic liver diseases characterized by abnormal lipid accumulation in liver [1–3]. According to recent epidemiological studies, the prevalence of NAFLD is 25.24% worldwide and 27.37% in the Asia-Pacific region [4], while the prevalence in China is in the range of 24.47%–29.70% [5]. Although NAFLD shows no obvious clinical manifestations in the early stage, it is an important risk factor for various cardiovascular diseases, type 2 diabetes mellitus, and other metabolic diseases [6–8]. It has been reported that the all-cause mortality risk was also significantly elevated in a NAFLD population [9, 10].

The pathogenesis mechanism of NAFLD is still vague except that insulin resistance (IR) and genetic predisposition are relatively confirmed [11–13]. Generally based on disease progression, NAFLD can be divided into three stages, beginning from non-alcoholic fatty liver (NAFL), then to non-alcoholic steatohepatitis (NASH), and, finally, liver cirrhosis, which is a much greater risk factor for primary liver cancer [1, 14]. Therefore, how to prevent disease progression from potentially reversible NAFLD and NASH to irreversible cirrhosis is the key point for the treatment [15]. Currently, there are still no confirmed interventions for NAFLD [16]. In view of this, many patients will seek medical help from Traditional Chinese Medicine (TCM) in China.

Based on TCM theory, NAFLD belongs to the category of “Gan Pi,” and “phlegm dampness” and “phlegm fluid retention” are its pathological products. The basic pathogenesis of NAFLD is transportation dysfunction of the spleen due to deficiency of spleen yang [17, 18]. The corresponding TCM pattern is spleen-yang deficiency. Main clinical manifestations include laziness to speak, poor appetite, loose stool, cold limbs, and so on. The basic treatment principle is warming yang to promote diuresis [19, 20].

Lingguizhugan decoction (LGZG), first documented in the ancient classic “Jingui Yaolue,” is a representative formula under the principle of warming yang to promote diuresis. It is composed of *Poria* (Fulin), *Ramulus Cinnamomi* (Guizhi), *Rhizoma Atractylodis Macrocephalae* (Baizhu), and *Radix Glycyrrhizae* (Gancao) [21]. Previous studies showed that LGZG can significantly decrease triglycerides (TG) and lipid contents of the liver tissue, and improve hepatic injury in high fat diet-induced NAFLD rats [20, 22]. LGZG was also reported to be capable of alleviating IR in patients with diabetes [23]. However, there is still no high-level evidence to support LGZG application the in treatment of NAFLD. In addition, the dose of LGZG still follows the classic. Dose optimization will also be clinically significant due to the variation of environment and lifestyle.

Moreover, with the development of intestinal microbiota, many studies have verified the role of intestinal microbiota in the pathogenesis of NAFLD. Regulation of intestinal flora can increase appetite and energy intake in food, regulate gene expression regarding fat synthesis and beta oxidation, and improve IR [24–26]. These mechanisms may be involved in the occurrence of NAFLD. Other studies also reported that most gut microbiota originate from the oral cavity, then speculated that oral microbiota are also correlated to NAFLD [27, 28]. TCM may play an important role in treating diseases through regulating microbiota. Therefore, it will also be valuable to explore the potential mechanisms of LGZG against NAFLD from the research on oral and intestinal microbiota.

Hence, we designed this study to evaluate the efficacy and safety of LGZG for NAFLD with a spleen-yang deficiency pattern and to assess the optimal dosage. The results of this trial may help to establish the evidence base for the application of LGZG in community healthcare centers. In addition, the research of oral and gut microbiota will benefit the exploration of potential mechanisms.

## Methods/design

### Study design

The study protocol was approved by the Medical Ethics Committee of Longhua Hospital Affiliated to Shanghai University of Traditional Chinese Medicine (2017LCSY069) and registered in the Chinese Clinical Trial Registry (http://www.chictr.org.cn/edit.aspx?pid=24555&htm=4) with number ChiCTR1800014364 on 1 January 2018.

**Fig. 1:**
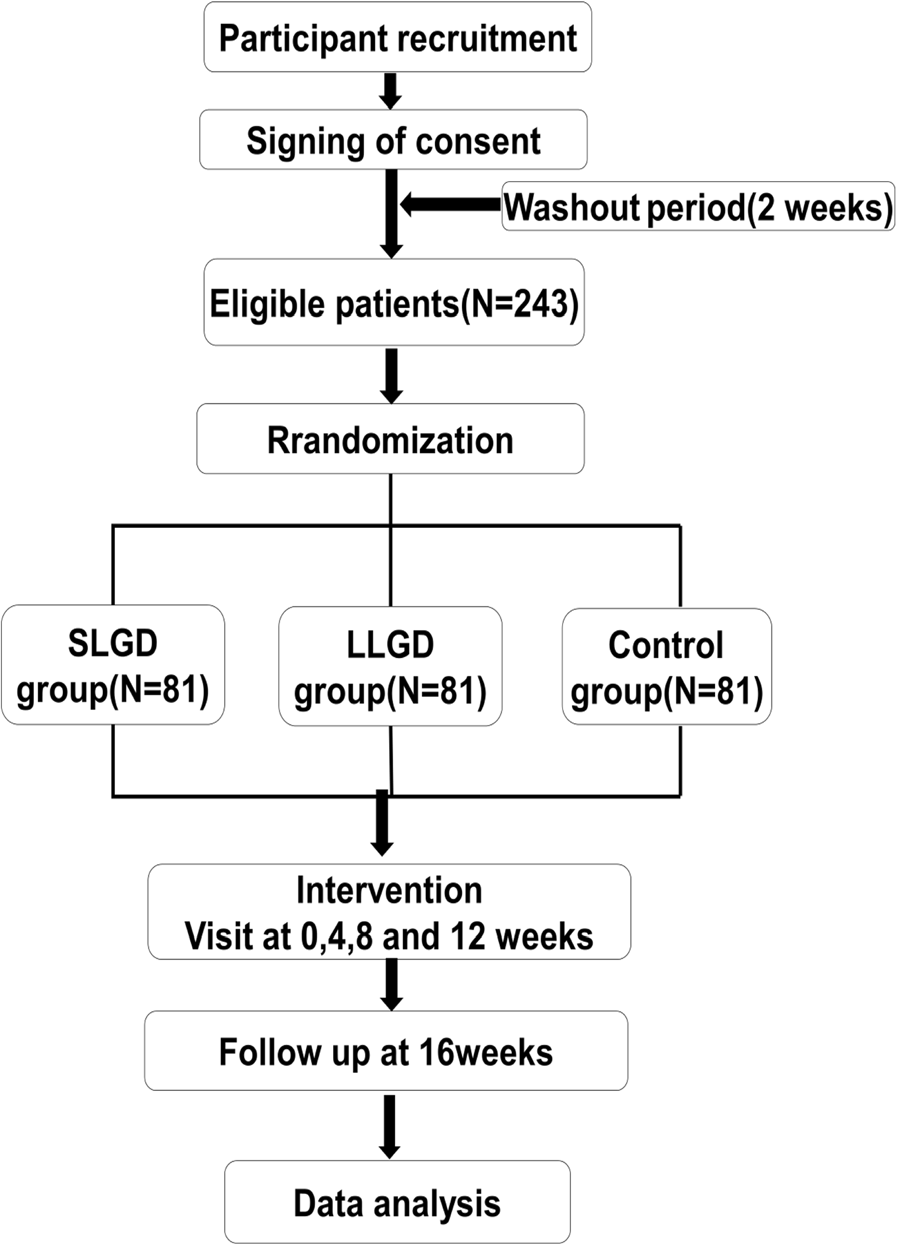
*Flow chart* of the clinical study

This study will be a 16-week, randomized, multi-center, double-blinded, three-arm, dose-optimization, placebo-controlled clinical trial. A total of 243 patients with NAFLD will be recruited through recruitment advertisements and introductions from doctors from three community healthcare centers in Shanghai, namely Zhangjiang community healthcare center, Beicai community healthcare center, and Sanlin community healthcare center. All three centers are based in Pudong New Area, Shanghai and no obvious difference will be supposed to exist in population and socioeconomic aspects. Eligible patients with NAFLD who agree to participate in the study will be randomly assigned to the standard dose Lingguizhugan decoction (SLGD) group, low dose Lingguizhugan decoction (LLGD) group, or the control group on a ratio of 1:1:1. On the basis of behavioral intervention therapy, patients in the SLGD and LLGD groups will be treated with different doses of LGZG; the control group will receive placebo. The LLGD group will be made of half of the standard dose, and the placebo will be one-tenth of the standard dose but with soluble starch, mixed colorant, and bitter component to achieve a comparable appearance, smell, and taste. The course of treatment will last 12 weeks. A 4-week follow-up will also be arranged to assess the continued effect. Participants will be assessed at week 0 (baseline), week 4, week 8, week 12 (end of treatment), and week 16 (end of follow-up). All participants will be asked to provide written informed consent before entering the trial. The study flow chart is shown in Fig. 1 and participant timeline is presented in Table 1. The Standard Protocol Items: Recommendations for Interventional Trials (SPIRIT) Checklist is presented in Additional file 1.

**Table 1:** Participant timeline

	Study phase					
	Screening	Allocation	Intervention			Follow-up
**Time point (weeks)**	**–2**	**0**	**4**	**8**	**12**	**16**
*Enrollment*						
Eligibility	**X**					
Informed consent	**X**					
**Allocation**		**X**				
*Intervention*						
SLGD			**X**	**X**	**X**	
LLGD			**X**	**X**	**X**	
Placebo			**X**	**X**	**X**	
*Assessment*						
Demographic indexes	**X**	**X**				
HOMA-IR		**X**	**X**		**X**	**X**
Hepatic function		**X**	**X**		**X**	**X**
Lipid metabolism		**X**	**X**		**X**	**X**
Glucose metabolism		**X**	**X**		**X**	**X**
Inflammation response		**X**	**X**		**X**	**X**
Liver-kidney echo ratio		**X**			**X**	
Body measurements		**X**	**X**	**X**	**X**	**X**
TCM pattern scale		**X**	**X**	**X**	**X**	**X**
SF-36		**X**			**X**	**X**
SAS		**X**			**X**	**X**
SDS		**X**			**X**	**X**
Vital signs		**X**	**X**	**X**	**X**	**X**
Blood routine test		**X**	**X**		**X**	**X**
Electrocardiograph		**X**	**X**		**X**	
AEs		**X**	**X**	**X**	**X**	**X**
Biological sample collection		**X**	**X**		**X**	**X**

*AE* adverse event, *LLGD* low dose Lingguizhugan decoction, *SLGD* standard dose Lingguizhugan decoction

### Participants

#### Diagnostic criteria

All participants should meet the diagnostic criteria of NAFLD and TCM pattern differentiation criteria of spleen-yang deficiency. Diagnostic criteria of NAFLD will be based on the American Association for the Study of Liver Diseases (AASLD) in 2017 [12]. To be specific, the following four criteria should all be met: (a) imaging (ultrasound, computed tomography, or magnetic resonance imaging) or histological evidence of hepatic steatosis; (b) no significant alcohol consumption, defined as > 21 standard drinks per week in men and > 14 standard drinks per week in women; (c) exclusion of other reasons inducing hepatic steatosis, including but not limited to hepatitis C, specific drugs induced, parenteral nutrition, and severe malnutrition; and (d) no coexistence of other chronic liver disease, including but not limited to hemochromatosis, autoimmune liver disease, chronic viral hepatitis, alpha-1 antitrypsin deficiency, hepatolenticular degeneration, and drug-induced liver disease.

The TCM pattern differentiation criteria of spleen-yang deficiency will refer to the expert consensus of spleen deficiency by China Association of Traditional Chinese Medicine in 2017, previous systematic review, and the clinical guidelines for the new Chinese medicine [29–31]. Ten symptoms and signs will be assessed by continuous 100-point scale, a higher score means a more severe degree. Every symptom or sign possesses with a certain weight. The scoring will be calculated through multiplying the rating score by the weight. Spleen-yang deficiency pattern will be defined as total score of 10 dimensions ≥ 20. The detailed rating scale is shown in Table 2.

**Table 2:** Spleen-yang deficiency pattern scale

Symptoms	Score (0–100)
Do you feel too lazy to speak?	20%
Do you sweat often or have night sweats even though you are not active and it is not hot?	5%
Do you have bland and tasteless feeling in your mouth?	10%
Does your stool have a thin texture and is loose?	20%
Do you feel more saliva?	10%
Do you have bleeding gums?	5%
Do you feel a lack of warmth in the extremities	10%
Do you have insomnia/trouble sleeping at night?	5%
Are you likely to catch a cold?	10%
Do these symptoms affect your eating habits (such as hate greasy good)?	5%
Total	

#### Inclusion criteria

Participants who meet all of the following criteria will be included: (a) aged 18–80 years, men or women; (b) confirmed diagnosis of NAFLD; (c) confirmed diagnosis of spleen-yang deficiency pattern; and (d) voluntary informed consent and agreement to participate in every visit, examination, and treatment according to the protocol.

#### Exclusion criteria

Participants who meet any of the following criteria will be excluded: (a) combination with other specific liver diseases which would induce fatty liver, including but not limited to alcoholic liver disease, chronic hepatitis C, autoimmune liver disease, and hepatocellular degeneration; (b) fatty liver induced by drugs (e.g. tamoxifen, ethylamine iodifurone, valproate, methotrexate, glucocorticoid), total parenteral nutrition, inflammatory bowel disease, hypothyroidism, Cushing syndrome, abetalipoproteinemia, and other syndromes related to insulin-resistance (e.g. lipid atrophic diabetes, Mauriac syndrome); (c) combination with serious primary diseases and mental diseases, including but not limited to cardiovascular and cerebrovascular diseases, hepatic diseases, renal diseases, hematologic diseases, cancers, and schizophrenia; (d) combination with diabetes or currently receiving anti-diabetic medicine treatment; (e) currently receiving treatments for NAFLD (including Chinese herbal decoction, Chinese patent medicine, and chemical agents); (f) antibiotics administration in the last month; (g) allergy to compositions of experimental agents or possessing an allergic constitution; (h) pregnancy and lactating women, and women who are likely to be pregnant but refuse to keep predefined contraception measures during the study; (i) participation in other clinical trials in the last 3 months or currently joining other trials; (j) mental or legal disability; (k) cannot obey medical advice for therapeutic lifestyle modifications; and/or (l) suspicious of drug abuse or possessing other forbidden criteria.

### Interventions

All clinical investigators responsible for diagnosis and treatment will be registered practitioners of TCM. Before the start of the trial, a 3-day training session will be held for all clinical investigators involved in the study. All participants with NAFLD will receive treatment for 12 weeks according to the random assignment. LGZG has been utilized for a long time in clinical practice. Participation in this trial will not anticipate bringing any harm to the patients. Hence, no post-trial care will be arranged.

#### Behavioral intervention

Behavioral interventions will be given to all three groups and the prescription will be based on the Guidelines for Prevention and Control of Overweight and Obesity in Chinese Adults [32]. In brief, participants will be required to limit calorie intake (approximate 1660 kcal per day) and guarantee physical exercises (moderate aerobic exercise at least 150 min per week). Individuals will be asked to complete daily dietary and exercise records during treatment (Additional file 2).

#### Drug intervention

**Fig. 2:**
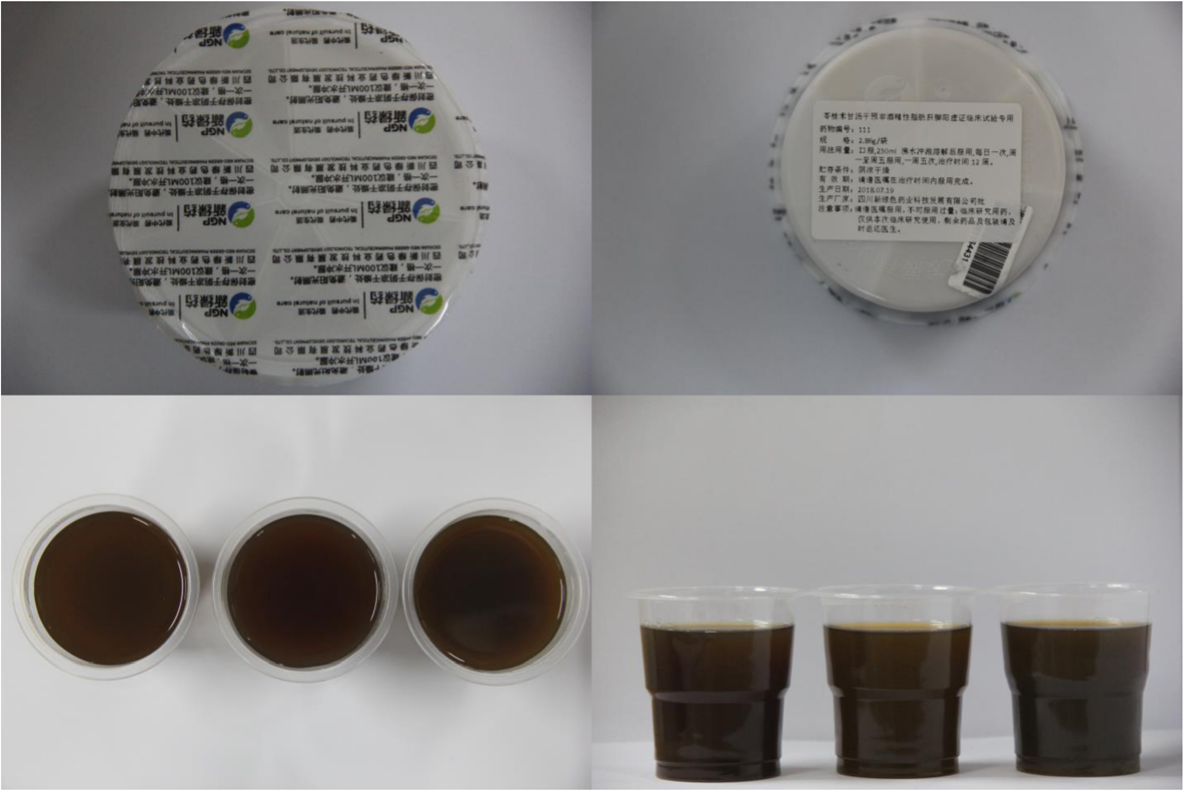
Appearance of drug samples from three groups

LGZG is a traditional formula which is composed of *Poria* (Fulin), *Ramulus Cinnamomi* (Guizhi), *Rhizoma Atractylodis Macrocephalae* (Baizhu), and *Radix Glycyrrhizae* (Gancao). The standard dose for each herb is 12 g, 9 g, 6 g, and 6 g, respectively. The LLGD group will be made of half of the standard dose and the placebo will be one-tenth of the standard dose. The course of treatment lasts 12 weeks. Participants will be required to take one dosage (one pack) of granules 30 min after breakfast, once daily on weekdays. A total of 60 packs per patient will be administrated. The granules used in this study will be produced by Sichuan Neo-green Pharmaceutical Technology Development Co., Ltd. (Sichuan, China). The crude herbs will be extracted and made into granules. The production process will be under the standard of Good Manufacturing Practice. No significant difference was found among granules samples from the three groups in terms of color, appearance, shape, smell, and weight (Fig. 2).

#### Concomitant treatment regulations

Except for research products, other drugs (including Chinese herbal decoctions, Chinese patent drugs, and chemical drugs) and interventions (including acupuncture, cupping, and massage) that may interfere with the trial will be forbidden during the study. For participants with other existing diseases who cannot stop relevant therapies should record concomitant interventions in detail. If the disease progresses during the study period, patients can withdraw from the study and use other treatment methods. The case will be regarded as invalid and the patient will be required to complete the relevant examinations and evaluations as much as possible.

#### Drug dispensation and compliance assessment

Drugs will be packed as required in the double-blinded study. During the treatment period, a certain amount of packs will be distributed in week 0, week 4, and week 8 (22 packs). The two additional packs will be reserved for unforeseen circumstances. After taking the medicine, the patients will be asked to return all the remaining products and packages at their next visit. The clinical investigators will count the number of remaining products and calculate the participants’ compliance. The research team will establish a WeChat group for health guidance and periodically contact individuals by telephone or WeChat to check safety and improve compliance.

### Outcome measures

The schedule for all outcome measures is shown in Table 1. All demographic data will be determined before treatment, including the date of birth, gender, body weight, height, medication history, past medical history, smoking and drinking status, and course of disease.

The primary outcome will be the proportion of participants with at least a 1-unit decrease of HOMA-IR from baseline to treatment endpoint (week 12) [33]. Secondary outcomes will include body measurements (body weight and body mass index), hepatic function (alanine aminotransferase [ALT], aspartate transaminase [AST], gamma-glutamyl transpeptidase [GGT], and alkaline phosphatase [ALP]), lipid metabolism profiles (total cholesterol [TC], TG, low-density lipoprotein cholesterol [LDL-C], high-density lipoprotein cholesterol [HDL-C], apolipoprotein A [apoA], apolipoprotein B [apoB], and non-high-density lipoprotein cholesterol [non-HDL-C]), glucose metabolism profiles (fasting blood glucose [FPG], fasting insulin [FINS], and glycosylated hemoglobin [HbA1c]), inflammatory responses (white blood cell [WBC] counts, C-reactive protein [CRP], and inflammatory cytokines), liver-kidney echo ratio by ultrasound, and scorings of various questionnaires (TCM spleen-yang pattern scale, the MOS item short from health survey [SF-36], self-rating depressive scale [SDS], and self-rating anxiety scale [SAS]). All secondary outcomes will also be evaluated from baseline to treatment endpoint.

Safety variables will include vital signs (including blood pressure, respiratory, heart rate, and body temperature) evaluated at every visit, electrocardiogram (ECG) and blood tests (including blood routine test and renal function) evaluated at baseline, week 4, and week 12. Adverse events (AEs) will be observed and recorded throughout the whole trial. LGZG is a classical herbal formula and has satisfactory safety profiles. Potential AEs may be mild gastrointestinal reactions, which will be alleviated spontaneously without any additional medical interventions. Clinical investigators should document any AEs in a case report form (CRF), including the following information: occurrence time; symptoms and signs; severity; duration; laboratory examinations; interventions; outcomes; and follow-up time. If an AE occurs, clinical investigators should adopt appropriate measures to guarantee the safety of participants based on the actual situation. If serious AEs occur, clinical investigators should inform the principal investigator within 2 h of the event. The principal investigator should then report to the Medical Ethics Committee and Data Monitoring Committee (DMC) within 24 h. It will be decided whether to terminate the trial after a discussion.

### Biological sample collection

In order to further explore the mechanism of LGZG on NAFLD and its effect on oral and gut microbiota, biological samples will be collected in the present study. On the consent form, the collection of biological samples will be clearly stated and the participants will have the right to refuse to provide biological samples. All biological specimens will be used for auxiliary researches in the future. Blood samples will be planned for subsequent DNA methylation and metabonomics studies. Feces and coating on the tongue will be planned for gut and oral microbiota studies. Whole blood, morning urine, feces, and coating on the tongue will be collected at baseline, 4 weeks, 12 weeks, and 16 weeks. All samples will be transferred and stored in a refrigerator at 80 °C within 24 h.

### Sample size estimation

Sample size was estimated based on the clinical primary outcome. As far as we know, this is the first RCT evaluating the efficacy of LGZG versus placebo. No data are available as a reference. Therefore, based on our previous animal studies and consultation with TCM experts, we assumed that the effect size for the SLGD group is 30%, LLGD group is 15%, and placebo group is 10%. If α = 0.05 and β = 0.10, the sample size can be calculated by the following formula:

$$ \mathrm{n}=\frac{1641.4\lambda }{{\left({\sin}^{-1}\sqrt{p_{max}}-{\sin}^{-1}\sqrt{p_{min}}\right)}^2} $$

The λ is the degree of freedom, taking 12.65 in this case. The p_max_ is 30% and the p_min_ is 10%. Assuming the maximum dropout rate is 20%, 81 patients will be required for each group, with a total of 243 patients.

### Randomization and blinding

All participants will be randomly allocated to the SLGD group, LLGD group, or control group based on a ratio of 1:1:1. The random number table will be generated by SPSS 19.0 for Windows software. A research assistant who will not participate in this clinical trial will send the random number table to the pharmaceutical company for packaging to maintain the blinding of participants and clinical investigators. According to the order of enrollment, the clinical investigators will assign the numbers in ascending order and the pharmacists will distribute the corresponding drug. Participants, clinical investigators, and statisticians will all be blinded until completion of the whole trial. The randomization list and blinding codes will be kept strictly confidential.

Based on the requirements of medical ethics, a double-blind study should set up an emergency letter for each blind number. Emergency letters will be sealed in opaque envelopes. The contents of the letter will include group details and emergency situations. Emergency letters should be sealed and can only be opened when necessary. The emergency letter will be read by clinical investigators in cases of severe AEs, in which knowing the experimental treatment is compulsory for further medical interventions. The case will be treated as a withdrawal and the clinical investigator will record the corresponding reason in the CRF. All emergency letters will be retrieved together with the CRF after the study. Double unblinding will be used in this trial. Briefly speaking, the first unblinding will uncover the codes of treatments, namely A, B, and C. After the completion of the statistical analysis, the second unblinding will uncover the specific drugs corresponding to the cords.

### Data collection and management

The Trial Steering Committee will be composed of the principal investigator (GJ) and coordinators from three centers. The committee will be responsible for revising study design, reviewing overall progress and communicating with Medical Ethics Committee per 6 months. An independent DMC will also be established by the trial steering committee. The DMC will include clinical epidemiologists, data monitors, and statisticians. No competing interest will exist. The DMC will review the efficacy and safety data according to the participants’ schedule and provide suggestions to the Trial Steering Committee on revising the study design or terminating the trial.

Clinical investigators will be responsible for the data collection during this study according to the schedule. CRFs should be filled in after every visit to guarantee the accuracy and timeliness. Data monitors from the DMC will review the CRFs each month. When a participant finishes all treatments and follow-up, the completed CRF will be verified by the coordinator. During the research process, the Trial Steering Committee will also assign research assistants every month from the Shanghai University of Traditional Chinese Medicine to solve potential problems encountered during the trial and guarantee the quality of the study execution.

In order to improve the compliance of patients to the intervention, free tests and free medical education will be provided. On the consent form, participants will be informed that they can withdraw the trial at any time and that the withdrawal decision will not interfere the regular medical care. Participants will also notice that their personal information will be de-identified throughout the trial to guarantee confidentiality. For participants who discontinue or deviate from intervention protocols, the researcher should contact the individuals, ask them for reasons, record the last time of taking medicine, and complete the evaluation items as much as possible.

### Statistical analysis

The analysis of efficacy and safety will be based on the principle of intention-to-treat (ITT), namely all randomized participants will enter the final analysis. The missing value will be filled by the method of the last observation carried forward. Data will be presented as mean with standard deviation, median with range, or number with percentage. Data analysis will use the SPSS 19.0 for Window software. All statistical inferences will be performed using a two-sided test with a statistically significant test level of *P* < 0.05 and the confidence interval of parameters will be estimated by 95% confidence interval. Differences within groups will be assessed by paired t-test, chi-square test, or Wilcoxon signed rank test according to specific variables. Differences between groups will be assessed by one-way analysis of variance or Kruskal–Wallis test based on characteristics of data. Post-hoc pairwise multiple comparisons will be performed if statistical significance is found.

No interim analysis was arranged in this trial due to a short intervention period and safety profiles of LGZG. Potential sub-group analyses will be determined based on characteristics of included participants, for instance, different categories of body mass index and different combined diseases.

### Publication and dissemination

Regardless of the efficacy of drugs, the results will be disseminated through publications. The future report of this clinical trial will follow the requirements of CONSORT statement [34] and Chinese herbal medicine Formulas extension [35]. Authorship for the final report will be determined based on the contributions to the trial. We will not intend to use professional writers.

## Discussion

NAFLD is a widely health problem and > 80% of obese people suffer from NAFLD globally [36]. A large population-based study has demonstrated that NAFLD is one of the important causes of hepatocellular carcinoma (HCC) and the incidence of NAFLD-related HCC is increasing by approximately 10% per year [37]. However, the pathogenesis mechanism of NAFLD is vague [38]. There is still no drug currently approved for the treatment of NAFLD by the U.S. Food and Drug Administration (FDA) or the European Medicines Evaluation Agency (EMEA) [10, 39]. Hence, many patients with NAFLD will seek help from TCM interventions, especially Chinese herbal medicine.

Treatment based on pattern differentiation is the character of understanding and treating disease by TCM. Therefore, we introduce pattern differentiation as one of inclusion criteria for NAFLD participants, namely spleen-yang deficiency. LGZG is the corresponding formula for this pattern. Previous animal studies also indicated the potential efficacy of LGZG against NAFLD [20, 22]. A clinical trial will then be necessary to demonstrate the application. To our knowledge, our trial is the first multi-center, randomized, double-blinded, placebo-controlled clinical trial to assess the efficacy and safety of LGZG for NAFLD.

To ensure the quality of this research, a strict system of training and supervision will be established. Training sessions will be held for each center to explain the study protocol and potential upcoming matters. How to confirm the TCM pattern of spleen-yang deficiency and how to collect biological samples will also be explained in training sessions. The biochemical measurements of each participating center will be tested uniformly by KingMed Diagnositics (Shanghai) to guarantee the consistency. The ECG and liver-kidney echo ratio by ultrasound will be examined in the corresponding experimental center with uniform standard.

The results of this trial will provide initial evidence regarding the efficacy and safety of LGZG in the treatment of NAFLD with spleen-yang deficiency pattern. Biological samples collected in this trial may explain the possible action mechanisms of LGZG associated with oral and gut microbiota.

## Trial status

We recruited participants from July 2018 and completed in February 2019. All clinical data were locked in June 2019. This protocol was submitted before completion of recruitment.

## Supplementary information

**Additional file 1.** SPIRIT 2013 checklist.

**Additional file 2.** Daily dietary and exercise records.

## Data Availability

Because no datasets were analyzed or generated during the present study, data sharing does not apply to this article. The data generated in this clinical trial will be available for non-commercial scientific researches after completing all data analysis and de-identification. Application should include the relevant research aim and methods, and be sent to the Trial Steering Committee. The data sharing decision will be made after discussion.

## Abbreviations

AASLD: American Association for the Study of Liver Diseases
AEs: Adverse events
ALP: Alkaline phosphatase
ALT: Alanine aminotransferase
apoA: Apolipoprotein A
apoB: Apolipoprotein B
AST: Glutamic pyruvic transaminase
CRF: Case report form
CRP: C-reactive protein
ECG: Electrocardiogram
EMEA: European Medicines Evaluation Agency
FDA: Food and Drug Administration
FINS: Fasting insulin
FPG: Fasting blood glucose
GGT: Gamma-glutamyl transpeptidase
HbA1c: Glycosylated hemoglobin
HCC: Hepatocellular carcinoma
HDL-C: High-density lipoprotein cholesterol
IR: Insulin resistance
ITT: Intention-to-treat
LDL-C: Low-density lipoprotein cholesterol
LGZG: Lingguizhugan decoction
LLGD: Low-dose Lingguizhugan decoction
MOS: Medical Outcomes Study
NAFL: Non-alcoholic fatty liver
NAFLD: Non-alcoholic fatty liver disease
NASH: Non-alcoholic steatohepatitis
non-HDL-C: Non-high-density lipoprotein cholesterol
SAS: Self-rating Depressive Scale
SDS: Self-rating Anxiety Scale
SF-36: MOS item short from health survey
SLGD: Standard dose Lingguizhugan decoction
SPIRIT: The Standard Protocol Items: Recommendations for Interventional Trials
TC: Total cholesterol
TG: Triglycerides
TCM: Traditional Chinese medicine
WBC: White blood cell
