# Supplementary material for: Traditional Chinese medicine Lingguizhugan decoction treating non-alcoholic fatty liver disease with spleen-yang deficiency pattern: Study protocol for a multicenter randomized controlled trial
Source: Trials. 2020 Jun 10;21:512. doi: 10.1186/s13063-020-04362-7 (PMC7288405; doi:10.1186/s13063-020-04362-7)
Supplement: Supplementary file 2 — Additional file 2. Daily dietary and exercise records. [file 13063_2020_4362_MOESM2_ESM.docx]

**Additional file 2.** Daily diet and exercise record chart

| **Meal times** | **Food name** | **Raw material name** | **Raw material weight**  **(Per 50 grams)** | **Raw material code** |
| --- | --- | --- | --- | --- |
| Breakfast |  |  |  |  |
|  |  |  |  |  |
|  |  |  |  |  |
|  |  |  |  |  |
| Lunch |  |  |  |  |
|  |  |  |  |  |
|  |  |  |  |  |
|  |  |  |  |  |
|  |  |  |  |  |
|  |  |  |  |  |
|  |  |  |  |  |
|  |  |  |  |  |
| Dinner |  |  |  |  |
|  |  |  |  |  |
|  |  |  |  |  |
|  |  |  |  |  |
|  |  |  |  |  |
|  |  |  |  |  |
|  |  |  |  |  |
| Other |  |  |  |  |
|  |  |  |  |  |
|  |  |  |  |  |
|  |  |  |  |  |
| Nutritional supplement |  |  |  |  |
|  |  |  |  |  |
|  |  |  |  |  |
|  | **Type of exercise** | | **Exercise Duration** | |
| Exercise situation |  | |  | |
|  |  | |  | |
|  |  | |  | |
